# Incidence of Emergency Department Presentations of Symptomatic Stone Disease in Pediatric Patients: A Southeastern Study

**DOI:** 10.7759/cureus.30979

**Published:** 2022-11-01

**Authors:** Shirley Y Zhang, Joshua D Collingwood, Ayaka Fujihashi, Kai He, Lauren A Oliver, Pankaj Dangle

**Affiliations:** 1 Department of Research, University of Alabama at Birmingham Heerskink School of Medicine, Birmingham, USA; 2 Department of Research, Alabama College of Osteopathic Medicine, Dothan, USA; 3 Department of Research, University of Alabama at Birmingham Heersink School of Medicine, Birmingham, USA; 4 Pediatric Urology, University of Alabama at Birmingham, Birmingham, USA; 5 Pediatric Urology, Indiana University, Indianapolis, USA

**Keywords:** calciuria, kidney, urinary stones, emergency department, kidney stones, pediatrics, nephrolithiasis

## Abstract

Background

The incidence of nephrolithiasis during childhood has increased significantly over recent decades. Some studies indicate a rapid rise in adolescents, particularly in African American women. This study serves to identify trends in symptomatic pediatric nephrolithiasis presentations to the emergency department (ED) as a result of increasing incidence and to determine associations between demographic variables at our single-site tertiary pediatric hospital in the Southeast United States.

Methods

After IRB approval, a review of the data provided by the Pediatric Health Information System, a pediatric database that includes clinical and resource utilization data for 51 of the largest children’s hospitals in the nation, yielded 644 pediatric occurrences of nephrolithiasis at single-site emergency departments from 2006 to 2020. The percent change and average percent change in three-year intervals were calculated to establish a trend over time. A chi-square test of independence was performed to assess associations between race, gender, and age groups.

Results

A total of 780 stone occurrences and associated patient demographic data were reviewed for 644 children (364, 56.52% female) with median age of 183 ± 45.11 months (9-397 months). Of the 644 children, 79 (12.3%) were noted to have recurrent symptomatic nephrolithiasis, contributing to 136/780 stone events. There was a marked increase of 84.4% in confirmed pediatric nephrolithiasis occurrences over 15 years, with an average percent increase of 16.1% every three years. A Chi^2^ test of independence was performed between gender and age group (>/< 10yr), gender and race, and race and age group. No expected cell frequencies were less than five. There is no statistically significant relationship between gender and age group, χ^2^ (1, *N*=644) = 3.30, *p*=0.692. There is no significant association between race (Caucasian vs. non-Caucasian) and age group (>/< 10yr), χ^2^ (1, *N*=644) = 0.393, *p*=0.531. There is a statistically significant relationship between gender and race (Caucasian vs. non-Caucasian), χ^2^ (1, *N*=644) = 5.28, *p*=0.021. Caucasian females were more likely to present to our tertiary pediatric hospital’s emergency department with nephrolithiasis than Caucasian males or non-Caucasian males or females. Additionally, our data reflected a greater percentage of symptomatic nephrolithiasis presentations occurred in the second decade of life (85.4% vs 14.3%, 552 vs 92 stone events).

Conclusion

Based on our data, there is a marked increase of 84.4% in pediatric nephrolithiasis occurrences from 2006 to 2020, with a mean increase of 16.1% every three years at our single-site tertiary referral pediatric hospital in the Southeast. Among demographic groups, white adolescent females have an increased risk of developing kidney stones.

## Introduction

The prevalence of nephrolithiasis in children has risen dramatically in recent decades [1,2,3]. Estimates of the contemporary mean annual incidence of pediatric nephrolithiasis range from 36 to 57 per 100,000 children in US population-based observational studies [2, 4]. Routh et al. assessed epidemiological trends in pediatric nephrolithiasis using the Pediatric Health Information System, identifying an adjusted annual increase of 10.6% in nephrolithiasis cases between 1999 and 2008 [2]. A recent study identified a larger increase in nephrolithiasis among 15-19-year-olds, with a 26% increase in incidence every five years from 1997 to 2012 [5]. Though the etiology of this increasing incidence is unknown, the morbidity associated with nephrolithiasis is cause for concern in the pediatric population. Another factor for consideration is the financial burden associated with it. Pediatric nephrolithiasis is estimated to cost the United States approximately $375 million annually in hospital and emergency department (ED) costs [6]. Sturgis et al. conducted a cost comparison of conservative to surgical management of pediatric nephrolithiasis, finding the proportion of conservatively managed cases increased from 2011 to 2018, with a parallel in increasing costs of non-operative management across all treatment settings over that same period [7]. This study serves to identify trends in symptomatic pediatric nephrolithiasis to ED as a result of increasing incidence and to determine associations between demographic variables at our single-site tertiary pediatric hospital in the Southeast United States. This article was previously presented as a meeting abstract at the American Urological Association's Annual Meeting in 2022, held on May 14, 2022.

## Materials and methods

A retrospective study was conducted at Children's of Alabama, a tertiary pediatric hospital in Birmingham, Alabama, with a large referral base from the entire state. The study was approved by the office of the Institutional Review Board (IRB) at the University of Alabama at Birmingham (160923001).

Inclusion and exclusion criteria

The eligible population comprised all symptomatic patients ≤18 years of age who had been evaluated at Southeast tertiary pediatric hospital’s emergency department (ED) and confirmed to have had their first occurrence of nephrolithiasis by abdominal x-ray, ultrasound, or computed tomography scan between January 1, 2006, and January 1, 2020. Symptomatic presentation is defined by presentation with severe abdominal/flank pain, nausea, vomiting, fever >100.4ºF, and/or hematuria. Patients with asymptomatic and incidental stone diagnoses during an emergency department visit, as well as patients with missing data, were excluded. 

Data collection

Study data was provided by the Pediatric Health Information System (PHIS). The PHIS is a comparative pediatric database that includes clinical and resource utilization data for inpatient, ambulatory surgery, emergency department, and observation for patient encounters for 51 children’s hospitals. The PHIS hospitals are 51 of the largest and most advanced children's hospitals in America and constitute the most demanding standards of pediatric service in America.

Statistical analysis

The percent change and average percent change in three-year intervals were calculated to establish a trend over time. The Chi2 test of independence was employed to compare categorical variables and assess relationships. A p-value of 0.05 was considered statistically significant. All data analyses were conducted by IBM Statistical Package for Social Sciences (SPSS) Statistics.

## Results

A total of 780 stone occurrences and associated patient demographic data for 644 children (364, 56.52% female) with a median age of 183 ± 45.11 months (9-397 months) were reviewed. Of the 644 children, 79 (12.3%) were noted to have recurrent symptomatic nephrolithiasis, contributing to 136/780 stone events. Patient demographic variables and nephrolithiasis presentations to the ED in three-year increments are provided in Table 1. 

**Table 1:** Demographic characteristics of 644 pediatric patients with nephrolithiasis

Variables		n = 644
			Demographic data (n, %)		
	Female gender	364 (56.52)
	Median age (month, Q1, Q3)	183 (145, 205)
	< 10 years old	90 (14)
	> 10 years old	554 (86)
Race (n, %)		
	White	546 (84.78)
	African American	78 (12.1)
	Hispanic	18 (2.8)
	Asian	1 (0.16)
	Indian American/Alaskan	1 (0.16)
Cases every three years		
	2006-2008	90
	2009-2011	145
	2012-2014	171
	2015-2017	208
	2018-2020	166

**Figure 1:**
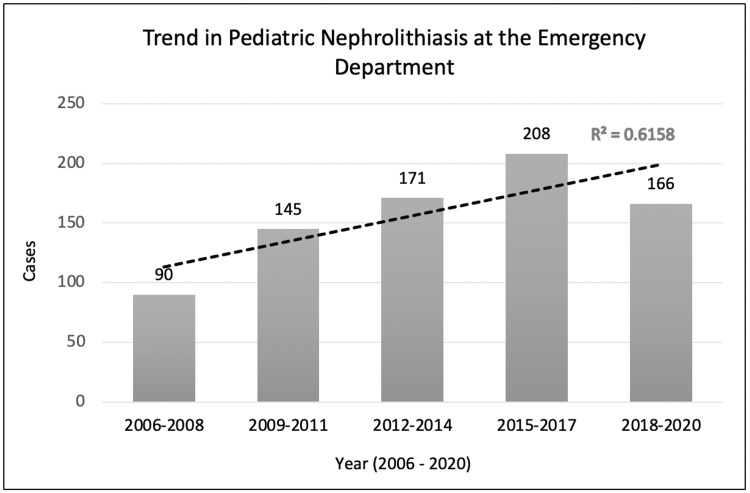
Trend in pediatric nephrolithiasis at the emergency department

There was a marked increase of 84.4% in confirmed pediatric nephrolithiasis occurrences over 15 years, with an average percent increase of 16.1% every three years. Additionally, a decline in nephrolithiasis presentation was identified from 2018 to 2020 (Figure 1).

A Chi2 test of independence was performed between gender and age group (>/< 10yr), gender and race, and race and age group. No expected cell frequencies were less than five. There is no statistically significant relationship between gender and age group, χ2 (1, N=644) = 3.30, p=0.692. There is no significant association between race (Caucasian vs. non-Caucasian) and age group (>/< 10yr), χ2 (1, N=644) = 0.393, p=0.531.

There is a statistically significant relationship between gender and race (Caucasian vs. non-Caucasian), χ2 (1, N=644) = 5.28, p=0.021. Caucasian females were more likely to present to our tertiary pediatric hospital’s emergency department with nephrolithiasis than Caucasian males or non-Caucasian males or females. Additionally, our data reflected a greater percentage of symptomatic nephrolithiasis presentations occurred in the second decade of life (85.4% vs 14.3%, 552 vs 92 stone events).

## Discussion

Increasing evidence suggests that pediatric nephrolithiasis is increasing in prevalence nationwide [1,2,3]. A retrospective series from Schneider Children’s Hospital in New York reported a five-fold increase in the number of children diagnosed with nephrolithiasis from 1994 to 2005 [8]. Similarly, emergency department data from South Carolina demonstrated a marked increase in the incidence of pediatric nephrolithiasis between 1996 and 2007 [4]. Our results corroborate these findings, with our single-site tertiary referral pediatric hospital’s ED seeing a marked increase of 84.4% in pediatric nephrolithiasis cases over the 15-year study period.

A published case series indicates nephrolithiasis is historically more common in boys than in girls [9]. However, recent population-based studies now demonstrate a significant rise in girls, with the discrepancy in gender distribution becoming more evident in adolescent populations [4,9,10]. Our findings demonstrate that Caucasian girls are more likely to present to the ED with symptomatic nephrolithiasis, with an increased risk in adolescence (>10 years of age). While our findings support a relationship between gender and age group, our study found little evidence to support a relationship between race and gender or race and age group. Further studies are necessary to determine how these factors contribute to the increasing incidence of pediatric nephrolithiasis.

The increasing incidence of pediatric nephrolithiasis has both direct and indirect costs. Literature has shown that as pediatric nephrolithiasis becomes more prevalent among children, there will be an increased economic burden placed on employers as well as on government resources [6]. A study assessing the nationwide economic impact of pediatric nephrolithiasis, evaluating both inpatient costs and costs associated with ED presentation, found the median charge per pediatric admission was $13,922, with a total estimated national inpatient cost of $229,636,006 in 2009. The median captured ED charges were $3,991 per encounter for a total cost of $146,700,230 nationally in that same year. When combining inpatient and ED charges, the estimated hospital-based charge for pediatric nephrolithiasis in 2009 was approximately $375 million [6]. A recent study found outpatient management accounted for 52% of overall spending (vs. 32% in inpatient management) in pediatric nephrolithiasis and that, despite increasing costs in outpatient management, children with nephrolithiasis are more likely to be seen in an outpatient setting [7]. Additionally, the contribution of outpatient management and missed child and parent-school and work days are likely to be substantial, though little data is available to bring clarity to the degree of contribution. For this reason, the $375 million should be considered a conservative estimate of the true economic burden of pediatric nephrolithiasis.

This study has several limitations, including the retrospective study design and the collection of data from a single center. Because of the differences in the characteristics of the patient population, treatment, and prescribing patterns in the community, these findings may not be generalizable. Since the study was retrospective and only included symptomatic presentations to our emergency department, it is possible that not all patients with nephrolithiasis were referred to our institution. Additionally, due to the retrospective nature of this study, the exact stone location was not available for analysis. The quality and quantity of studies on pediatric nephrolithiasis remain limited, and few recent studies exist. Many symptomatic patients who consulted outside hospital physicians may have been treated conservatively and not captured in the database. Much of the evidence for clinical treatment is derived from adult literature. Further studies are necessary to determine regional and national trends as well as demographic risk factors for pediatric nephrolithiasis.

## Conclusions

With this increasing trend in pediatric nephrolithiasis presentations to the ED, it is important for pediatric urologists to engage not only with primary care physicians in the community but also with our emergency medicine physicians to recognize the risk factors for symptomatic nephrolithiasis and identify the contributors to this increasing incidence.
